# Benign Cystic Mesothelioma of the Peritoneum Arising at the Greater Omentum in a 14-Year-Old Boy

**DOI:** 10.70352/scrj.cr.24-0085

**Published:** 2025-05-01

**Authors:** Yutaka Hirayama, Naruki Higashidate, Kengo Nakaya, Yasushi Iinuma

**Affiliations:** Department of Pediatric Surgery, Niigata City General Hospital, Niigata, Niigata, Japan

**Keywords:** mesothelioma, abdominal mesothelioma, benign cystic mesothelioma, calretinin, omental tumor

## Abstract

**INTRODUCTION:**

Mesothelioma arises from mesothelial cells. This tumor is very rare among pediatric abdominal neoplasms. We herein report an extremely rare case of acute abdomen with cystic mesothelioma of the peritoneum in a child.

**CASE PRESENTATION:**

A 14-year-old boy was referred to our hospital for emergency surgery. Surgery revealed a primary tumor arising from the greater omentum. The tumor was macroscopically diagnosed as a benign omental lymphangioma and was resected en bloc with the greater omentum. A histopathological examination of the tumor revealed a simple columnar epithelium-like mesothelioma with poor cell-atypia. Immunohistochemical examination showed antibody reactivity in the cyst epithelium, including an anti-calretinin antibody. The final pathological diagnosis was a mesothelioma originating from the peritoneum.

**CONCLUSIONS:**

In pediatric cases diagnosed with cystic lymphatic malformation, the possibility of peritoneal mesothelioma needs to be considered and carefully confirmed or ruled out.

## Abbreviations

CRP
: C-reactive protein
WBC
: white blood cell

## INTRODUCTION

Mesothelioma arises from mesothelial cells that cover most parts of the body. Most mesotheliomas involve the pleura in association with reactive mesothelial proliferation; however, rare cases involve the peritoneum in the pelvic cavity.^1)^ Peritoneal mesothelioma in a child is particularly rare, and differentiating abdominal mesothelioma from lymphangioma using imaging is exceptionally difficult^1,2)^ (**Table 1**). In such cases, deciding on an appropriate therapeutic strategy can be challenging.

**Table 1:** The pediatric omental mesothelioma cases (<16 year old)

Year	Author	Age (years)	Sex	Chief complaint	Preoperative diagnosis	Suspected origin	Treatment	Cofirmed origin
1991	Pollack CV Jr^2)^	15	F	Abdominal pain	Multilocular cystic mass	Pelvis	Total excision	Within the omentum
2025	Our case	14	M	Abdominal pain	Cystic lymphangioma	Mesentery	Total excision	At the greater omentum

We herein report the extremely rare case of an acute abdomen with cystic mesothelioma of the peritoneum arising at the greater omentum in a child.

## CASE PRESENTATION

**Fig. 1:**
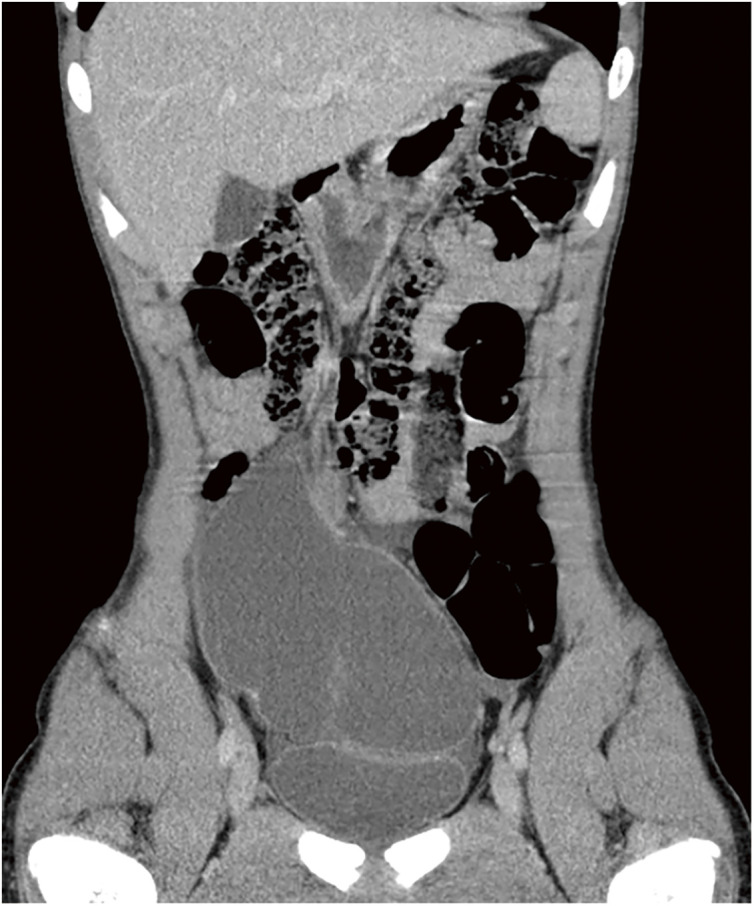
Computed tomography revealed a multicystic lesion in the right ileocecal region and ascites fluid in the lateral paracolic gutter.

A 14-year-old boy with a fever and right-sided abdominal pain was referred to our hospital. He had a history of Asperger syndrome but no history of drug treatment or exposure to asbestos. After the onset, the patient gradually adopted a more anteflexed position, and his previous doctor suspected acute appendicitis. Marked inflammation was confirmed by blood biochemistry, including a white blood cell (WBC) count of 20900/μL, neutrophil (Neut.) proportion of 88.3%, and C-reactive protein (CRP) level of 5.82 mg/dL. Computed tomography revealed a multicystic lesion in the right ileocecal region and ascites fluid in the lateral paracolic gutter; therefore, a bacterial infection complicating cystic lymphangioma of the small bowel mesentery was suspected (**Fig. 1**).

**Fig. 2:**
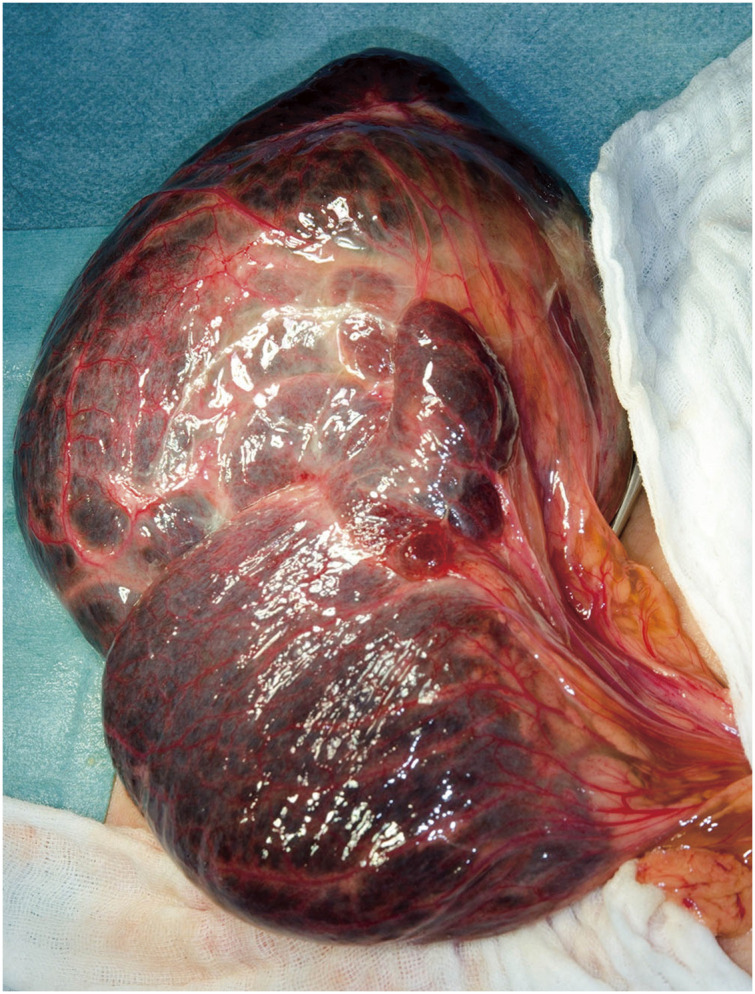
This surgery revealed the primary tumor arising from the greater omentum. The cystic tumor had also invaded much of the right lower abdomen that rode on the transverse colon.

However, as the possibility of peritonitis due to cyst rupture could not be excluded, emergency surgery was planned for the diagnosis and radical treatment of the primary tumor. Surgery revealed a primary tumor arising from the greater omentum. The cystic tumor had also invaded much of the right lower abdomen, while also invading the transverse colon (**Fig. 2**). Scattered cystic lesions and invasion into the surrounding tissues were not evident. The tumor was thus macroscopically diagnosed as a benign omental lymphangioma, and was resected en bloc with the greater omentum.

**Fig. 3:**
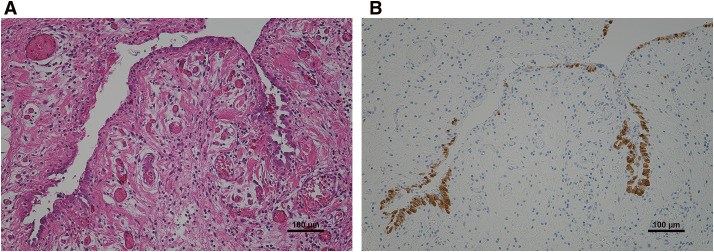
(**A**) Histopathological examination showed a double-layered structure with vascular hyperplasia and high infiltration of inflammatory cells. (**B**) Immunohistochemical examination showed antibody reactivity on the cyst epithelium, including for anti-calretinin antibody.

The tumor weighed 555 g with dimensions of 150 mm × 100 mm. No fulfilling part was apparent in the lumen, which contained only the serous fluid. The wall structure of the tumor was densely packed with cysts of various sizes and composed of fibrous components or fatty tissue. A histopathological examination revealed a double-layered structure with vascular hyperplasia and high infiltration of inflammatory cells, with a layer of gelatin between the inner and outer membranes of the capsule (**Fig. 3A**). The luminal surface was lined with simple columnar epithelium-like mesothelioma with poor cell-atypia. A immunohistochemical examination showed antibody reactivity in the cyst epithelium, including anti-calretinin, anti-CK 5/6, anti-WT-1, and anti-D2-40 antibodies. The final pathological diagnosis was a mesothelioma originating in the peritoneum (**Fig. 3B**).

Ten years postoperatively, no findings suggestive of recurrence or complications were observed in the patient.

## DISCUSSION

Mesothelioma arises from membranous tissues consisting of mesothelial cells, such as the pleura, pericardium, peritoneum, and testicular membrane.^1)^ In particular, mesothelioma originating in the peritoneum was reported by Plaut^3)^ in a case involving multiple peritoneal cysts. Subsequently, Mennemeyer et al.^4)^ described a clearly defined strategy for diagnosing the histopathological findings of multicystic mesotheliomas. In adult cases, approximately 63% of patients have a history of chronic inflammation inside the pelvic cavity, such as open surgery or endometriosis.^5)^ By contrast, pediatric cases without a history of open surgery, such as the present case are extremely rare.^6,7)^

In terms of etiology, a connection between reaction theory and long-term stimulation of the involved membrane is conventionally supported.^8)^ However, lymph node metastasis or malignant transformation is also observed in rare instances, suggesting the involvement of unknown mechanisms beyond chronic inflammation.^9,10)^ Particularly in pediatric patients, the possibility of a mechanism based on membrane proliferation as low-grade or borderline malignancy should be considered.^11)^ In addition, tumor theory and pathogenesis of malignant mesothelioma are unrelated in cases with a history of asbestos.^12)^

In terms of the clinical picture, most patients with benign cystic mesothelioma of the peritoneum (about 80%) are women around 37 years old.^1,13)^ At this point, it is very interesting to compare the age of onset with the characteristic features of cystic lymphatic malformations. Lymphatic malformations are more common in men than in women, and more than 50% of such cases are <5 years old.^14)^ Furthermore, symptoms in cases of peritoneal mesothelioma derive from compression and traction by the growing tumor, and thus are likely to be absent in the early stages.^8,15)^ In the present case, the patient was also likely aware of pain in the right lower abdomen during position changes and abdominal compression as the cystic component grew larger.

Although preoperative diagnostic imaging can provide an excellent definition of cystic lesions with a thin bulkhead, a more detailed analysis of the components is believed to be difficult.^16)^ In the present case, the identification of the primary focus could not be achieved preoperatively. A definitive diagnosis of this disease is only possible through a pathological examination of a surgical specimen. Identifying the epithelium comprising a monolayer of squamous or cuboidal cells is important, as is confirmation of positive immunohistochemical findings for mesothelial cell markers, such as anti-calretinin, anti-WT-1 antibody, anti-cytokeratin5/6 antibody, anti-AE1/AE3, anti-D2-40, anti-HBME-1, anti-mesothelin, and anti-thrombomodulin antibody.^8,12)^ Such positive findings are imperative for proving the mesothelial origin (**Fig. 3B**). Therefore, if a child presents with an abdominal cystic disease that tends to increase in size, a histopathological evaluation after specimen extraction should be actively pursued.

Complete excision appears to be the most effective surgery.^1)^ In this case, resection proved uncomplicated because the tumor only arose from the omentum with no involvement of the retroperitoneum. However, other reports have suggested a close relationship between the presence of disseminated lesions and the rate of recurrence. For example, 88.9% of patients treated with open surgery showed free-floating cysts in intraperitoneal membrane tissue.^17)^ In pediatric cases diagnosed with cystic lymphatic malformation, although sclerotherapy using drugs or partial resection by surgery are mainly selected, if the cystic mass comprises multiple tiny lesions, the possibility of peritoneal mesothelioma needs to be kept in mind and carefully confirmed or ruled out.^13,17)^ For this purpose, a definitive diagnosis should be proactively pursued, such as a diagnosis together with a pathological examination during surgery.

## CONCLUSIONS

We encountered a pediatric case of an acute abdomen with cystic mesothelioma of the peritoneum arising in the omentum. This tumor is very rare among pediatric abdominal neoplasms. As this pathology does not shrink spontaneously, unlike lymphangioma, complete excision is required for radical treatment. We believe that the findings from the postoperative follow-up of this patient beyond 10 years suggests a low risk of recurrence, but many mechanisms remain unexplained about the natural history of cystic mesothelioma in the peritoneum of a child. Continued follow-up of this patient is important, even in adulthood.

## DECLARATIONS

### Funding

Not applicable.

### Authors’ contributions

All authors carried out the surgery and postoperatible management.

All authors have read and approved the final manuscript, and they are responsible for the manuscript.

### Availability of data and materials

Not applicable.

### Ethics approval and consent to participate

This work does not require ethical considerations or approval. Informed consent to participate in this study was obtained from the patient.

### Consent for publication

Oral informed consent was obtained from the patient for the publication of this case report and accompanying images.

### Competing interests

The authors declare that they have no competing interests.

## References

[1] Noiret B Renaud F Piessen G Multicystic peritoneal mesothelioma: a systematic review of the literature. Pleura Peritoneum 2019; 4: 20190024. 31667333 10.1515/pp-2019-0024 PMC6812218

[2] Pollack CV Jr Jorden RC. Benign cystic mesothelioma presenting as acute abdominal pain in a young woman. J Emerg Med 1991; 9(Suppl 1): 21–5. 1955676 10.1016/0736-4679(91)90582-z

[3] Plaut A. Multiple peritoneal cysts and their histogenesis. Arch Pathol 1928; 5: 754–6.

[4] Mennemeyer R Smith M. Multicystic, peritoneal mesothelioma: a report with electron microscopy of a case mimicking intra-abdominal cystic hygroma (lymphangioma). Cancer 1979; 44: 692–8. 476578 10.1002/1097-0142(197908)44:2<692::aid-cncr2820440242>3.0.co;2-6

[5] Ross MJ Welch WR Scully RE. Multilocular peritoneal inclusion cysts (so-called cystic mesotheliomas). Cancer 1989; 64: 1336–46. 2766227 10.1002/1097-0142(19890915)64:6<1336::aid-cncr2820640628>3.0.co;2-x

[6] Stojsic Z Jankovic R Jovanovic B Benign cystic mesothelioma of the peritoneum in a male child. J Pediatr Surg 2012; 47: e45–9. 10.1016/j.jpedsurg.2012.06.029 23084231

[7] Tuncer AA Narcı A Dilek FH Benign cystic mesothelioma in a child: case report and review of the literature. Balkan Med J 2016; 33: 232–4. 27403396 10.5152/balkanmedj.2015.15886 PMC4924971

[8] Khurram MS Shaikh H Khan U Benign multicystic peritoneal mesothelioma: a rare condition in an uncommon gender. Case Rep Pathol 2017; 2017: 9752908. 28607791 10.1155/2017/9752908 PMC5451755

[9] Engohan-Aloghe C Anaf V Noël JC. Lymph node involvement in multicystic peritoneal mesothelioma. Int J Gynecol Pathol 2009; 28: 594–7. 19851213 10.1097/PGP.0b013e3181aae8f6

[10] Rapisarda AMC Cianci A Caruso S Benign multicystic mesothelioma and peritoneal inclusion cysts: are they the same clinical and histopathological entityes? A systematic review to find an evidence-based management. Arch Gynecol Obstet 2018; 297: 1353–75. 29511797 10.1007/s00404-018-4728-2

[11] González-Moreno S Yan H Alcorn KW Malignant transformation of 2 “benign” cystic mesothelioma of the peritoneum. J Surg Oncol 2002; 79: 243–51. 11920782 10.1002/jso.10081

[12] Weiss SW Tavassoli FA. Multicystic mesothelioma. An analysis of pathologic findings and biologic behavior in 37 cases. Am J Surg Pathol 1988; 12: 737–46. 3421410

[13] Alvir I Bevanda B Danolić D Benign multicystic peritoneal mesothelioma mimicking gynecologic pathology. Acta Clin Croat 2021; 60: 323–5. 34744286 10.20471/acc.2021.60.02.22 PMC8564840

[14] Carpenter HA Lancaster JR Lee RA. Multilocular cysts of the peritoneum. Mayo Clin Proc 1982; 57: 634–8. 7121070

[15] Yaegashi N Yajima A. Multilocular peritoneal inclusion cyst (benign cystic mesothelioma): a case report. J Obstet Gynaecol Res 1996; 22: 129–32. 8697341 10.1111/j.1447-0756.1996.tb00954.x

[16] Safioleas MC Constantinos K Michael S Benign multicystic peritoneal mesothelioma: a case report and review of the literature. World J Gastroenterol 2006; 12: 5739–42. 17007034 10.3748/wjg.v12.i35.5739 PMC4088182

[17] Yonemura Y Canbay E Sako S Multicystic mesothelioma has malignant potential: its grounds and mechanisms of peritoneal metastasis. J Peritoneum 2017; 52: 21–6.

